# Supplementary material for: Role and mechanism of NCAPD3 in promoting malignant behaviors in gastric cancer
Source: Front Pharmacol. 2024 Apr 22;15:1341039. doi: 10.3389/fphar.2024.1341039 (PMC11070777; doi:10.3389/fphar.2024.1341039)
Supplement: Supplementary file 11 [file DataSheet2.ZIP › GSEA/Transcipt factor/my_analysis.Gsea.1599461814734/FOXO3_01.html]

Details for gene set FOXO3\_01[GSEA]

|  || Dataset | filtered\_dataset.sample\_info.cls#WT\_versus\_NCAPD3\_MUT |
| Phenotype | sample\_info.cls#WT\_versus\_NCAPD3\_MUT |
| Upregulated in class | NCAPD3\_MUT |
| GeneSet | FOXO3\_01 |
| Enrichment Score (ES) | -0.4491371 |
| Normalized Enrichment Score (NES) | -2.1625772 |
| Nominal p-value | 0.0024875621 |
| FDR q-value | 0.040391825 |
| FWER p-Value | 0.227 |
Table: GSEA Results Summary

  

Fig 1: Enrichment plot: FOXO3\_01      
 Profile of the Running ES Score & Positions of GeneSet Members on the Rank Ordered List

  

| SYMBOL | TITLE | RANK IN GENE LIST | RANK METRIC SCORE | RUNNING ES | CORE ENRICHMENT || 1 | 23338 | JADE2 | 71 | 0.855 | 0.0114 | No |
| 2 | 1200 | TPP1 | 536 | 0.454 | -0.2899 | No |
| 3 | 4784 | NFIX | 567 | 0.439 | -0.2794 | No |
| 4 | 84335 | AKT1S1 | 572 | 0.437 | -0.2502 | No |
| 5 | 26100 | WIPI2 | 601 | 0.423 | -0.2395 | No |
| 6 | 149076 | ZNF362 | 680 | 0.388 | -0.2673 | No |
| 7 | 55122 | AKIRIN2 | 721 | 0.363 | -0.2696 | No |
| 8 | 31 | ACACA | 780 | 0.334 | -0.2869 | No |
| 9 | 9572 | NR1D1 | 977 | -0.392 | -0.3995 | No |
| 10 | 51667 | NUB1 | 978 | -0.392 | -0.3708 | No |
| 11 | 23764 | MAFF | 1039 | -0.436 | -0.3821 | No |
| 12 | 54541 | DDIT4 | 1133 | -0.502 | -0.4124 | Yes |
| 13 | 639 | PRDM1 | 1159 | -0.523 | -0.3920 | Yes |
| 14 | 27250 | PDCD4 | 1170 | -0.537 | -0.3599 | Yes |
| 15 | 2119 | ETV5 | 1176 | -0.545 | -0.3235 | Yes |
| 16 | 153222 | CREBRF | 1187 | -0.559 | -0.2898 | Yes |
| 17 | 10150 | MBNL2 | 1217 | -0.591 | -0.2674 | Yes |
| 18 | 54491 | FAM105A | 1241 | -0.619 | -0.2387 | Yes |
| 19 | 8408 | ULK1 | 1243 | -0.622 | -0.1938 | Yes |
| 20 | 3084 | NRG1 | 1320 | -0.739 | -0.1944 | Yes |
| 21 | 57157 | PHTF2 | 1334 | -0.755 | -0.1485 | Yes |
| 22 | 54463 | FAM134B | 1356 | -0.815 | -0.1039 | Yes |
| 23 | 4734 | NEDD4 | 1369 | -0.856 | -0.0498 | Yes |
| 24 | 26959 | HBP1 | 1395 | -1.073 | 0.0108 | Yes |
Table: GSEA details [plain text format]

  

Fig 2: FOXO3\_01      
 Blue-Pink O' Gram in the Space of the Analyzed GeneSet

  

Fig 3: FOXO3\_01: Random ES distribution      
 Gene set null distribution of ES for **FOXO3\_01**
